# Validation of Standing and Locomotion Scoring, Behavioral Assessments, and Mechanical Nociceptive Threshold Testing on Naturally Occurring Sow Lameness

**DOI:** 10.3390/ani13111801

**Published:** 2023-05-29

**Authors:** Anna K. Forseth, Locke A. Karriker, Suzanne T. Millman, Kenneth J. Stalder, Rebecca L. Parsons, Samaneh Azarpajouh, Anna K. Johnson

**Affiliations:** 1 Swine Medicine Education Center, College of Veterinary Medicine, Iowa State University, Ames, IA 50011, USA; 2 Veterinary Diagnostic and Production Animal Medicine and Department of Biomedical Sciences, College of Veterinary Medicine, Iowa State University, Ames, IA 50011, USA; 3 Department of Animal Science, College of Agriculture and Life Sciences, Iowa State University, Ames, IA 50011, USA

**Keywords:** lameness, swine, welfare, hoof, nociception, locomotion

## Abstract

**Simple Summary:**

One of the most common reasons that sows are removed from a farm when they would otherwise still be productive is from lameness. The causes of lameness are highly varied and could involve multiple body systems. Early detection, before the problem is untreatable, improves success. Several technologies have been developed to detect lameness earlier when it is less obvious; however, there has been limited testing of them on naturally occurring causes of lameness in sows. This study enrolled animals from a typical farm, moved them to an intensive study lab, and applied these tests to see if they were capable of accurately identifying a variety of naturally occurring lameness problems. Standing and moving lameness scoring, withdrawal from the application of pressure to the leg, the number of pig–human interventions needed to keep animals moving through an obstacle course, and the time to complete an obstacle course were evaluated. Standing and locomotion lameness scoring systems, mechanical pressure, and pig behavior could discriminate between animals with mild organic lameness and animals that were sound and may have utility on the farm and could be used by staff to identify and manage lame animals. In rare instances, the tools used here were able to discriminate between broad categories of lameness causes.

**Abstract:**

The objective of this study was to validate standing and locomotion lameness scoring, mechanical nociceptive threshold testing, and behavioral profile tools for the diagnosis of naturally occurring lameness etiologies in pigs. A total of 55 crossbred gilts and sows obtained from a commercial farm were enrolled in the study; with sound pigs classified as controls (8) and the remainder as lame due to integumentary (20), musculoskeletal (15), and combinations of integumentary and musculoskeletal (12) etiologies. Standing and locomotion lameness, mechanical nociceptive threshold (MNT) test, pig-human interventions, and latency to complete an obstacle course were evaluated. Standing and locomotion lameness scoring systems, MNT, and pig behavior (latency) were capable of discriminating between animals with mild organic lameness and animals that were sound and may have utility on the farm for staff to use to identify and manage lame animals. In rare instances, the tools used here were able to discriminate between broad categories of lameness etiology.

## 1. Introduction

In the production of animal agriculture, specifically swine, lameness has a substantial effect on animal welfare. The United States Department of Agriculture (USDA) reported that 8.5% of gilts and sows culled from the breeding herd were lame (USDA 2015) [1]. In a review of 19 international studies (Stalder et al., 2004) [2], the percentage of sows and gilts culled for lameness or locomotor problems ranged from 6.1 to 15.0% in these populations. This continual culling affects the economic return in the industry (Stalder et al., 2004) [2], worker morale (Deen and Xue, 1999) [3], and the individual pig’s welfare (Anil et al., 2009) [4]. In addition, it has been estimated that 32% of sows culled for lameness only produce one litter (Anil et al., 2005, Boyle et al., 1998) [5,6].

Identifying swine lameness objectively and early is crucial for successful on-farm management and therapeutic strategies. Previous work, using a chemically induced, transient, pig lameness model (Karriker et al., 2013) [7], successfully produced lameness of quantifiable severity using a variety of objective tools, including kinematics (Mohling et al., 2014a; Pairis-Garcia et al., 2015a) [8,9], mechanical nociceptive thresholds (Tapper et al., 2013; Mohling et al., 2014b) [10,11], and behavioral responses (Pairis-Garcia et al., 2015b; Parsons et al., 2015; Roca et al., 2016) [12,13,14]. Furthermore, induced lameness was ameliorated by non-steroidal anti-inflammatory drugs (NSAID) (Pairis-Garcia et al., 2013; Pairis-Garcia et al., 2014) [15,16]. Although these diagnostic tools have shown success in a research environment using an induced lameness model, there has been limited validation of them using naturally occurring sow lameness originating in a typical commercial production environment.

Subjective visual numerical rating scales (NRS) can be used on-farm at several time points throughout the production cycle (Manson and Leaver, 1988; Karriker et al., 2013) [7,17]. These NRS have been implemented to aid producers in quickly and affordably estimating lameness prevalence in swine (Main et al., 2000) [18] and other species (Sprecher, 1997) [19].

Laboratory-based validation of lameness detection tools relies on a narrow range of lameness causes, such as a chemically induced lameness model (Karriker et al., 2013) [7]. Data from farms are useful for understanding the economic impact; however, they broadly lump all causes of lameness together, which may not adequately inform or prioritize work on interventions. Typically, all lameness types are categorized under one generic category on-farm and are not uniformly evaluated by a veterinarian.

Examination by a trained veterinarian can associate locomotor disorders with specific systems or etiologies, including neurological etiologies, hoof or limb lesions, mechanical-structural problems, traumas, metabolic disturbances, and infectious diseases (Smith, 1988; Wells, 1984) [20,21]. It is plausible that lameness detection technologies do not perform consistently across different lameness etiologies. Therefore, this study combined the organic generation of lameness in a farm setting with the rigorous application of detection technology possible in the controlled environment of the laboratory for the objective of validating subjective lameness scoring, mechanical nociceptive threshold testing, and novel obstacle navigation tests relative to etiologies of naturally occurring lameness in pigs.

## 2. Materials and Methods

### 2.1. Animals and Housing

All procedures were approved by the Iowa State University Institutional Animal Care and Use Committee and met or exceeded the contemporary Guide for Care and Use of Agricultural Animals in Research and Teaching for the Care of Swine (FASS, 2010) [22].

Crossbred pigs were enrolled (gilts, *n* = 3; sows, *n* = 52) in the study as six groups, acquired over a three-month period. Pigs originated from a commercial farm that utilized individual stalls for breeding followed by group housing for the remainder of gestation. The breeding/gestation barn flooring was fully slatted concrete. Six visits were made to the same commercial farm over a three-month period. At each visit, the herd was evaluated until 9 or 10 pigs matched the enrollment criteria. Selected pigs displayed lameness, based on a standing lameness scoring system (Table 1) or gross limb abnormality. Animals that were non-ambulatory, presented with clinical signs of systemic disease other than lameness, or were non-weight bearing on a limb were excluded. The next day, after identification, pigs were transported approximately 60 min from the farm to the Swine Intensive Studies Laboratory at Iowa State University, Ames, IA. Once a group of 9–10 completed the study, a new group was identified at the farm and the process was repeated. The same evaluator conducted the herd evaluation and selected the pigs each time.

**Figure 1:**
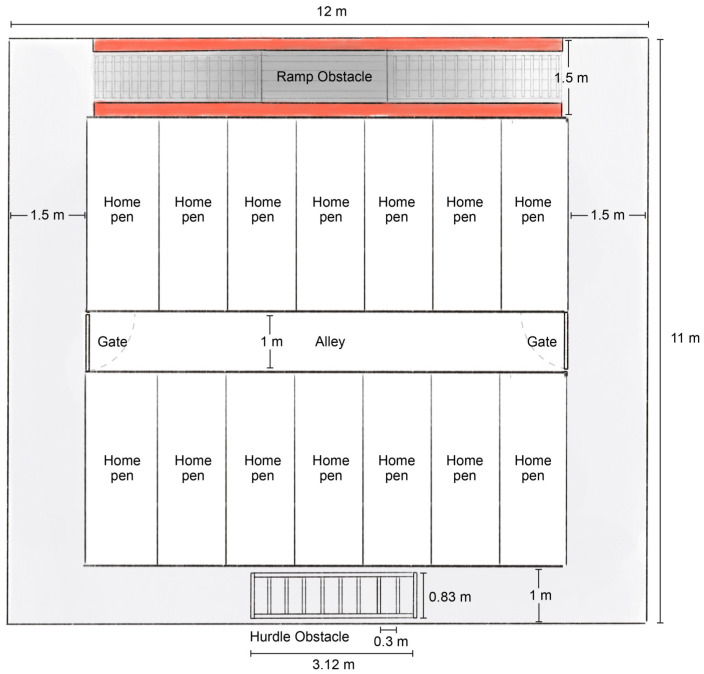
Schematic of the layout of the obstacle course that surrounded the home pens, indicating the location and size of obstacles for evaluating control (non-lame) and naturally occurring lame (lame) pigs. The obstacle course and pens were housed in a larger room (boundaries not indicated).

To avoid confounding injury due to aggression, each pig was housed individually in a concrete pen with 5.1 m^2^ of floor space. A rubber mat (FarmTekTM, Dyersville, Dyersville, IA, USA) was provided for comfort. Pens were configured in two rows with a central aisle, allowing for nose-to-nose contact between adjacent pigs but not across the aisle (Figure 1). Pigs were fed in a small feed bowl on a 0.6 m deep concrete ledge along the rear wall of the pen. All pigs were fed 2.2 kg of a commercial ration twice daily, which had been formulated to meet or exceed their dietary requirements (NRC, 2012) [23]. Pigs were provided ad libitum access to water via a one-nipple drinker that was positioned over a grate. Caretakers observed all pigs twice daily and verified that they were able to rise and were ambulatory on all four limbs. A photoperiod of 12:12 h light:dark cycle with light hours between 0600 and 1800. Pigs were acclimatized to the laboratory environment for one day, after which testing began.

### 2.2. Subjective Lameness Scoring

On test day, 24 h after arrival at the lab, standing lameness was scored in the pig’s home pen. Pigs were evaluated from outside the pen prior to feeding and in most cases, were standing spontaneously in anticipation of feed delivery. In cases where they were not, they were encouraged to stand with verbal and/or noise maker (rattle paddle) interactions from the caretakers. A single, trained, veterinarian made all lameness assessments. Pigs that no longer presented the lameness selection criteria previously observed at the sow farm (standing lameness and/or gross limb abnormality) were designated as non-lame controls. While each pig walked from their home pen to the test stall, their locomotion lameness score was evaluated (Table 1). A veterinarian (AF) identified the lame limb.

### 2.3. Mechanical Nociceptive Threshold Assessment

Mechanical nociceptive threshold was the minimum pressure that induced a reaction characteristic of pain or pain response. Pigs were individually assessed for mechanical nociception threshold (MNT) while confined in a modified gestation stall (0.6 m × 2.0 m) located outside the obstacle course. Feed was sprinkled into the stall feeder to facilitate standing in a relaxed posture (Mohling et al. 2014a; Pairis-Garcia et al. 2014) [8,9]. The MNT assessment was performed using a hand-held pressure algometer (PA; Wagner Force Ten FDX 50 Compact Digital Force Gage; Wagner Instruments, Riverside, CT, USA). The same technician performed all MNT assessments on all pigs during all test days. MNT was assessed on the lame limb and the corresponding limb on the opposite side (i.e., right rear lame compared to left rear sound). If lameness was not localized to a single limb, all four limbs were assessed. For control pigs, MNT was assessed on both limbs of either the right or left side, as selected by a coin toss.

Pigs were first desensitized by the application of slight pressure from the technician’s hands on the medial and lateral aspects of the limb, moving from the hock or elbow to the dewclaw. This process was completed until the pig did not react to pressure during two consecutive applications. The PA was held perpendicular to the limb, and the 1 cm^2^ flat rubber tip was applied 1 cm above the coronary band on the lateral aspect of the lateral claw, at a rate of 1 kg of additional force/s. The MNT (kgf) was defined as the point at which a withdrawal response was observed; at that time the PA was removed, and the peak pressure MNT value was recorded. To avoid bias, the technician was blind to the numeric output values applying PA, and a second technician recorded the data values. If there was no withdrawal the test was terminated at approximately 10 kg of force. The MNT test was repeated in triplicate on each limb selected for testing.

### 2.4. Obstacle Course

Following MNT assessments, the pig exited the modified gestation stall and entered the obstacle course. The obstacle course measured 45.0 m long × 1.5 m wide and included two obstacles designed to simulate common walking hindrances that pigs might encounter on-farm (Figure 1). Excluding the ramp, the course floor was covered with clean, solid gray, low pile, synthetic carpet, which was glued in place.

#### 2.4.1. Ramp Obstacle

**Figure 2:**
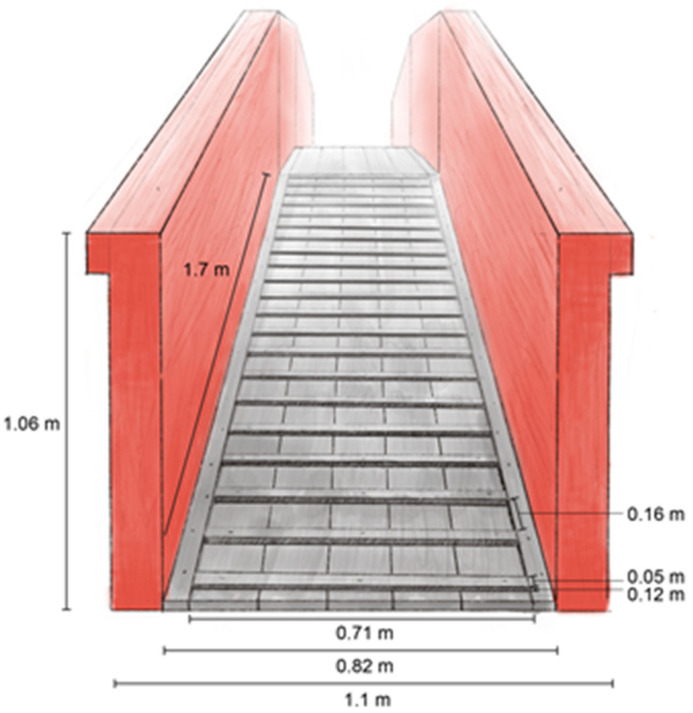
Schematic representation of the ramp obstacle with identical ascending and descending slopes and dimensions.

**Figure 3:**
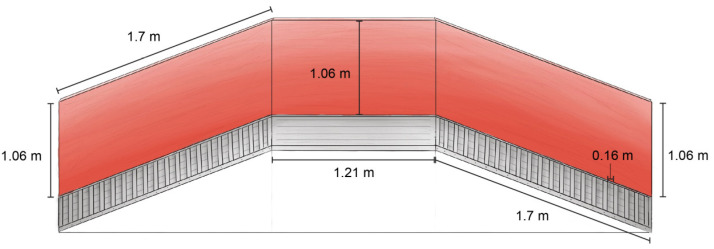
Schematic representation of the ramp obstacle from the side with nearest wall removed and dimensions indicated.

The ramp obstacle was constructed of wood, with ascending and descending slopes that were 171 cm long × 83 cm wide × 107 cm high (Figure 2). The ascending and descending ramp slopes were 11° and designed according to Transportation Quality Assurance (TQA) recommendations (NPB, 2020) [24]. The walkway was 122 cm long × 83 cm wide with 107 cm high side walls and connected the ascending and descending ramps (Figure 3). A total of 21 cleats, measuring 71 cm long × 5 cm wide × 3 cm high, were spaced at 17 cm intervals on both ascending and descending ramps (NPB, 2020) [24].

#### 2.4.2. Hurdle Obstacle

**Figure 4:**
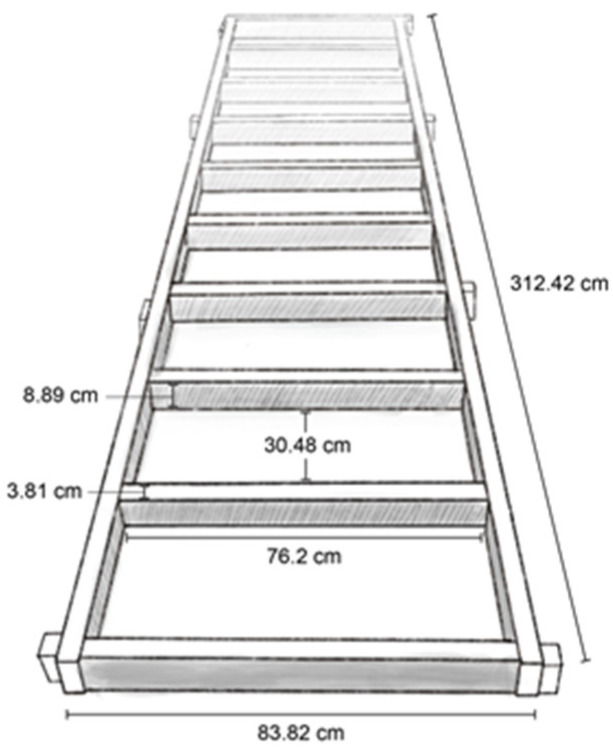
Schematic representation of the hurdle obstacle with dimensions indicated.

The hurdle obstacle was constructed to require the pig to lift and flex its limbs while stepping over the wooden boards (Figure 4). A total of 10 wooden boards measuring 9 cm high × 76 cm wide × 4 cm deep were spaced 30 cm apart were attached to a wooden frame. The wooden frame measured 312 cm long × 84 cm wide × 9 cm high.

### 2.5. Handling Interventions

Since the goal was to measure differences in movement and navigation of the obstacles, handling interventions were applied when the pigs stopped at various locations around the obstacle course. These interventions were standardized so that the number of interventions could indicate differences between the pigs’ reluctance or motivation and not the variability in their handling. Handling intervention was recorded when the pig stopped their forward movement for 10 consecutive seconds at any point during the obstacle course. Each intervention was applied in intervals lasting up to 20 s and involved 5 different levels: (1) noise from a plastic coffee can filled with metal pieces, (2) sorting panel pressure applied to the pig’s hindquarters/rump, (3) hand pressure applied to the pig’s back with verbal encouragement, (4) feed sprinkled on the ground in front of the pig and, (5) rattle paddle pressure applied to the pig’s back without striking. All interventions were applied consistently with TQA handling guidelines. We considered the five interventions to have increasing intensity levels. Handling intervention always began at level 1 and ceased when the pig moved forward. If the pig did not move forward, the next intervention level was applied. If a pig fell, sat, or laid down on the obstacle course, it was allowed to rest for 10 min without intervention. If a pig did not complete the obstacle course within 40 min, it was allowed to return to its home pen by the most direct route and nearest course exit, the associated course time was recorded as 40 min.

### 2.6. Video-Based Measurements

The outcomes of (1) the total number of handler interventions per obstacle and (2) the duration in seconds for pigs to traverse the obstacles were obtained by analyzing the video capturing the activities of the pig in the obstacle course. Five color cameras (Panasonic, Model WV-CP-484, Matsushita Co., LTD, Kadoma, Japan) were placed above the obstacle course; three cameras were positioned over the ramp obstacle and two cameras were positioned over the hurdle obstacle. The video was captured by utilizing two Noldus portable labs (Noldus Information Technology, Wageningen, NL, USA). Video recordings were collected at a speed of 10 frames/s and saved to a computer hard disk using HandiAvi (HandiAvi version 4.3 D, Anderson’s AZcendant Software, Tempe, AZ, USA).

Observations associated with handling interventions and track positions were collected from video recordings using continuous sampling, and all data were collected by one technician. All observations were performed using the Observer^®^ XT software program (version 10; Noldus Information Technology, Wageningen, NL, USA). Track positions defining the start and end of obstacles are described in Table 2. The technician collecting observations from the video was blinded to pig lameness status and etiology. A different researcher performed the blinding procedures for the video recordings for all tests. The blinding procedures involved cutting the video recordings to remove the identification process presented at the beginning of each video, assigning a random number to each video segment, and sorting for the purpose of providing a random sequence for the videos to be scored. Seven videos were selected at random and duplicated within this sequence for the purpose of determining intraobserver reliability. Prior to data collection, the observer was trained to use the Observer^®^ XT program by repeatedly scoring three videos from the ramp and three videos from the hurdle until reaching an acceptable interobserver reliability score (kappa > 0.8), as calculated by the program. After reaching the desired level of competence, data collection began using blinded videos. Intraobserver reliability was tested using seven clips from the ramp and eight from the hurdle, which were interspersed with the data collection.

### 2.7. Assessment of Suspected Etiology

Immediately after completion of the obstacle course, each pig was physically examined in their individual pen by a single veterinarian (AF). The initial physical examination included an evaluation of the pig’s alertness, responsiveness, and body condition. Limbs identified as lame, while the pig was standing and/or walking, were visually evaluated for function and to identify any skin abnormalities. As warranted by visual examination, limbs were also palpated to identify any soreness that may have been associated with deeper structures, including muscle and bone. The physical examination findings directed the veterinarian to one or more of the five body systems suspected to be involved in lameness, including (1) central nervous, (2) peripheral nervous, (3) digestive/metabolic, (4) musculoskeletal, and (5) integumentary.

### 2.8. Statistical Analysis

Each pig was considered an experimental unit. Of the 55 crossbred pigs enrolled in the study, 8 were classified as non-lame “controls” because both their standing and locomotion lameness score was 0 after transport and acclimation in the lab. Among the animals that were assessed as having a lameness score greater than 0 on either of the subjective numeric scoring systems, 3 suspected system etiologies were identified among the lame pigs (integumentary = 20; musculoskeletal = 15; integumentary and musculoskeletal = 12). The number of handling interventions within each type was insufficient to evaluate. Hence, all interventions were combined to create a total handling intervention score.

Duration to transverse the obstacles and handling intervention types were found to be correlated and were, therefore, analyzed separately using generalized linear mixed model methods (PROC GLIMMIX; SAS v9.4, SAS Inst. Inc., Cary, NC, USA). Durations for ramp incline, ramp walkway, ramp decline, total ramp obstacle, and total hurdle obstacle were transformed to log scale using the Gamma distribution option of the model statement.

The number of handler interventions by obstacle was summed for each pig regardless of level. Five models were developed for the number of handling interventions: ramp incline, ramp walkway, ramp decline, total ramp, and total hurdle. Handling intervention data were transformed to a log scale using the Poisson distribution option of the model statement. Fixed effects of group (*n* = 6), parity (0–7), suspected etiology (integumentary, musculoskeletal, and both integumentary and musculoskeletal), standing lameness score (0, 1), and walking lameness score (0, 1) were used for all models. The bodyweight (kg) for each pig was fit as a linear covariate.

For the mechanical nociceptive threshold model, the statistical design was a complete randomized design using a generalized linear mixed model (PROC GLIMMIX) with the model, including the fixed effects of pig BW (kg), suspected etiology, and the leg identified as lame. There were only 3 pigs displaying front leg lameness and, therefore, this was not included in the final data set. All three that demonstrated rear leg lameness, were classified as lame, and these data were included in the final data set.

Statistical differences were reported when individual model main effects were a significant source of variation (*p* ≤ 0.05). When individual model main effects were a significant source of variation, effect levels were separated using the PDIFF option. This provided the P values for the differences in the least squares means between the levels of fixed class effects. Results for fixed effects are reported as least squares means ± SE (LSMeans ± SE) after being back-transformed from the log scale using the ILINK option in the LSMEANS statement. Results for covariates are reported as regression coefficients ± SE.

## 3. Results

### 3.1. Assessment of Lameness and Suspected Etiology

There was no complete agreement between the locomotion and standing lameness assessments for the 34 pigs who scored as lame using the standing score and the 32 pigs who scored as lame using the locomotion score. Twenty-seven pigs were scored lame by both scoring systems. Of the 8 pigs designated “controls”, all were scored as 0 using both the standing and locomotion lameness scales in the laboratory evaluation and had no observable lesions or gross abnormalities, meaning that a lameness etiology was not assigned. Of the remaining, 20 were classified as integumentary, 15 as musculoskeletal, and 12 as integumentary/musculoskeletal. There were no lameness cases observed as being associated with the central nervous system, the peripheral nervous system, or the digestive/metabolic system.

### 3.2. Mechanical Nociceptive Threshold

When comparing the three lameness etiologies, lameness was not a significant source of variation for MNT, when the right (*p* = 0.55) or left (*p* = 0.36) hind limbs were lame and compared to the opposite leg. However, when ignoring the etiology source and comparing all the lame hind limbs versus all non-lame, there were differences in the MNT threshold scores for left (*p* = 0.02) and right (*p* = 0.06) lame hind limbs.

### 3.3. Obstacle Course

The standing lameness score was a significant source of variation (*p* < 0.01) when comparing the duration to transverse the incline portion of the ramp obstacle, whereby the non-lame pigs took longer to traverse the incline portion of the ramp obstacle than the lame animals. (*p* < 0.01; Table 3). The locomotion lameness score was a significant source of variation in the duration to transverse the incline-, walkway, and total ramp, as well as the hurdle obstacle, whereby the lame pigs were slower than the non-lame pigs (*p* < 0.05; Table 3). The standing and locomotion lameness scores were not significant sources of variation when evaluating the number of handler interventions required for the ramp and hurdle obstacles (*p* ≥ 0.05; Table 4).

Lameness etiology was a significant source of variation when comparing the duration to transverse the ramp walkway (*p* = 0.03), as well as the total ramp (*p* = 0.02). Control pigs took longer to cross the ramp walkway, and control and musculoskeletal pigs took longer to traverse the entire ramp (Table 5). This was also reflected in the number of handler interventions applied to encourage the transversion of the ramp obstacle (Table 6). Musculoskeletal and/or control pigs required the highest number of interventions in all three phases of the ramp.

## 4. Discussion

When designing this study, we expected that the transportation of lame pigs from a farm to a controlled laboratory environment and subjecting them to an acclimation period, while withholding therapeutic intervention, had the potential to increase the severity of their lameness. This would have meant that the pigs less accurately represented early and mild lameness, for which we were attempting to validate detection tools to identify. The decision to limit the acclimation period to 24 h reflected this concern. However, the effect of transportation and acclimation provided the opposite effect, whereby eight of the pigs no longer tested lame using the same standing lameness scoring that was used to select them on the farm. Owing to the shipment logistics from the farm, and the 24 h acclimation period, at least 36 h passed from the assessment at the sow farm until the first assessment at the lab. There are several potential explanations for this. It is possible that their lameness score was due to discomfort from the environment, such as the floor texture at the sow farm, or that the presence of mild inflammation had resolved itself over the subsequent 36 h. Since the sows were not accompanied by an extensive case history at the time of selection, it is possible that some were already well past peak lameness and in the process of resolution. To reduce the impact of variability created by the shipment and acclimation procedures, we elected to repeat the assessment at the start of the lab phase and classify sows at that point.

The obstacles evaluated in this study were designed to both mimic situations commonly found on farms. The ramp obstacles mimicked the trailer loading chute, while the wooden board obstacle mimicked the changes in the flooring when moving between locations on the farm. Our expectation was these would provide a more severe challenge to the pigs that were judged lame by either standing or locomotion scoring systems and would require more time to traverse, thereby requiring a greater number of handler interventions. Notably, however, the standing and locomotion scoring agreed that 27 of the pigs were lame and 8 of the pigs were not lame, meaning that the two systems disagreed on 20 of the pigs. When the pigs were classified using the locomotion scoring system, the lame group took longer to traverse both the ramp and board obstacles, as expected. When classified using the standing scoring system, animals classified as lame traversed the incline portion of the ramp obstacle faster than the non-lame animals. This incongruity between the subjective numerical rating scales has implications on farming as the sow and gilt housing is rapidly transitioning from gestation stalls to gestation pens, which allow animals to be observed while walking. This change will potentially facilitate more accurate detection of lameness using locomotion scoring rather than standing scoring systems.

We expected that lame pigs would uniformly require more time and intervention to move through obstacles in their path in all situations but that was not what we observed. Possible explanations may include: (1) the pain associated with locomotion for lame pigs may cause more focus on the path in front of them and less distraction by the environment around them, (2) the lame pigs may have reacted quicker to handler interventions due to a heightened sensitivity from pain, and lastly, (3) once removed from their individual pen, lame pigs may have been more motivated to return to their individual pens where they were previously not being asked to move.

In the present study, non-lame pigs took longer and required more handler interventions to enter the ramp incline and traverse the walkway. These findings may be explained by some non-lame pigs spending more time investigating their environment than the lame pigs. A future research consideration may include performing behavioral observations in the pigs’ original farm environment where novel stimuli to explore is less likely to be encountered.

While the laboratory obstacles were designed to simulate common obstacles encountered on commercial sow farms, there are many on-farm environmental factors that could not be replicated. These factors included noises, such as those from other pigs and boars and farm equipment–for example, feed augers, power washers, as well as smells, and sights, that may influence the speed a pig travels from point A to B. A better understanding of these aforementioned environmental factors and how they influence the pigs’ willingness to move from point A to point B, when lame or not lame, needs further investigation.

The results from this study confirm the effectiveness of MNT to detect lameness but also highlight limitations when using MNT to discriminate between lameness etiologies. There were two factors that may have influenced the results from MNT. Firstly, the measurement method, which in this study was the PA, where pressure was applied at the focal point and was consistently located on the limbs of all pigs (Nalon et al., 2016) [25]. Secondly, the specific anatomical location evaluated can influence the pig’s withdrawal response (Nalon et al., 2016) [25]. For example, we would expect response variation to the pressure applied near the pig’s coronary band with osteochondrosis at the stifle, compared to a pig with an injury on the distal limb. Additionally, the injury location can influence the pig’s desire to withdraw the affected limb, arbitrarily heightening the amount of pressure tolerated by the pig.

In general, we found less variation in performance on any of the lameness measurements between the various lameness etiologies. There are several potential explanations including that the differential effects of lameness due to the different etiologies or mechanisms do not manifest until lameness is more severe than evaluated in this study. Perhaps external evaluation, in the absence of more advanced veterinary tools, such as radiographs, is not refined enough to accurately classify etiology.

Future studies of this type would be well served to consider how to objectively differentiate slower movement through an obstacle course or pathway, which is due to curiosity or interaction with the environment and latency to move consistently with lameness. In our professional veterinary practice, in the field, we note that often when sows are moved between locations on the farms, practices have evolved to avoid “driving” the animals in favor of establishing a pathway with gates and letting the animals explore the path. Anecdotally, when we observed pigs moving through the obstacle course in this trial, they would often stop and investigate the environment, although it was relatively sparse compared to that on an active farm. For example, we vacuumed the track each day to eliminate dust, dirt, manure, and spilled feed.

## 5. Conclusions

The multifactorial nature of naturally occurring lameness makes determining the contributing etiology and body systems difficult. Ultimately, the suspected system needs diagnostic confirmation. The results from the present study should be carefully applied because the results may not appropriately predict the time and intervention needed to move pigs with differing etiologies and lameness severity. In conclusion, the results from the present study identified that walking and standing lameness scoring systems and pig behavior are promising tools for a producer to use on commercial pig farms to distinguish between lame and non-lame pigs.

It is important to recognize that this study was complicated by the necessity to move naturally occurring lame animals from the farm environment to the intensive study lab. For this reason, the selection of the original lame animals was biased toward milder lameness that would still allow for the pig to be transported humanely within current guidelines. Hence, the magnitude of differences reported here is likely underestimated compared to more severe lameness and should be extrapolated with caution. However, pigs with relatively milder lameness represent a subpopulation on the farm that is most likely to respond favorably to intervention relative to more severe or advanced lameness cases and that is why we focused on that population.

## Figures and Tables

**Table 1:** Ethogram for determining standing lameness and locomotion lameness scores of pigs with naturally occurring lameness.

Score	Description
Standing lameness	
0	Equal weight bearing on all 4 limbs and no toe tapping
1	Pig displayed any of the following: abnormal stance defined as a slightly arched back with lowered head, difficulty standing but bearing weight on all four legs, the affected lame leg bearing less weight or toe tapping
Locomotion lameness	
0	Pig did not appear lame during walking
1	Pig presented stiff, ataxic, displayed a swaying gait, had a shortened stride, or had a visible limp. Pig had some difficulty with locomotion or displayed a moderate kyphotic posture

**Table 2:** Position for determining intervals to transverse obstacles by non-lame (control) and naturally occurring lame (lame) pigs.

Obstacle	Description
Ramp obstacle	
Incline	Shoulders or hindquarters are positioned over the incline of the ramp
Walkway	Front hooves and shoulders or hind hooves and hindquarters are positioned over the walkway
Decline	Shoulders or hindquarters are positioned over the decline of the ramp
Total	Shoulders are positioned over the incline of the ramp until hindquarters leave the decline of the ramp
Hurdle obstacle	Front legs and shoulders are positioned over the first wooden board until hind legs and hindquarters leave the final wooden board

**Table 3:** Duration(s) to transverse obstacles by non-lame (control) and naturally occurring lame (lame) pigs relative to subjective lameness scores (*p* ≤ 0.05).

	Scoring System					
	Standing			Locomotion		
Obstacle	Lame	Non-Lame	*p*-Value	Lame	Non-Lame	*p*-Value
No. Pigs	34	21		32	23	
Ramp obstacle						
Incline	20 ± 3.0	45 ± 8.4	0.008	53 ± 10.2	17 ± 3.0	0.006
Walkway	238 ± 5.4	40 ± 12.6	0.24	70 ± 23.4	13 ± 3.6	0.003
Decline	70 ± 13.2	106 ± 15.6	0.87	114 ± 18	95 ± 14.4	0.50
Total	155 ± 19.2	191 ± 27	0.32	233 ± 36	127 ± 18	0.02
Hurdle obstacle						
Total	40 ± 4.8	41 ± 5.40	0.95	57 ± 8.4	29 ± 4.2	0.008

**Table 4:** Number of handler interventions applied to encourage the transversion of obstacles by non-lame (control) and naturally occurring lame (lame) pigs relative to subjective lameness scores (*p* > 0.05).

	Scoring System			
	Standing		Locomotion	
Obstacle	Lame	Non-Lame	Lame	Non-Lame
No. Pigs	34	21	32	23
Ramp obstacle				
Incline	2	1	1	2
Walkway	4	3	3	4
Decline	9	8	9	7
Total	15	12	13	13
Hurdle obstacle				
Total	2	2	2	3

**Table 5:** Duration(s) to transverse obstacles by non-lame (control) and naturally occurring lame (lame) pigs relative to their suspected etiologies.

	Suspected Etiology				
Obstacle	Control	Integumentary	Musculoskeletal	Integumentary/Musculoskeletal	*p*-Value
No. Pigs	8	20	15	12	
Ramp obstacle					
Incline	35.4 ± 12.6	20.4 ± 3.6	42.0 ± 10.8	27.0 ± 7.2	0.11
Walkway	117.0 ± 61.8 ^a^	20.4 ± 6.0 ^b^	20.4 ± 8.4 ^b^	14.4 ± 6.0 ^b^	0.03
Decline	109.2 ± 31.2	85.2 ± 12.26	145.2 ± 30.6	88.2 ± 18.6	0.27
Total	247.2 ± 66 ^a^	124.2 ± 17.4 ^b^	234.6 ± 45.6 ^a^	122.4 ± 23.4 ^b^	0.02
Hurdle obstacle					
Total	56.4 ± 15.6	35.4 ± 4.2	35.4 ± 6.6	40.2 ± 8.4	0.43

^a,b^ Different superscripts within a row indicate significant differences (*p* < 0.05).

**Table 6:** Number of handler interventions applied to encourage transversion of the ramp obstacle by non-lame (control) and naturally occurring lame (lame) pigs relative to suspected etiologies.

	Suspected Etiology				
Obstacle	Control	Integumentary	Musculoskeletal	Integumentary/Musculoskeletal	*p*-Value
No. Pigs	8	20	15	12	
Incline	13.1 ± 1.80 ^a^	6.8 ± 0.69 ^b^	20.9 ± 2.23 ^c^	9.1 ± 1.02 ^a^	0.05
Walkway	4.1 ± 1.26 ^a^	1.4 ± 0.34 ^b,c^	2.1 ± 0.51 ^a,c^	1.5 ± 0.40 ^b,c^	0.05
Decline	6.5 ± 1.11 ^a^	4.8 ± 0.54 ^a^	11.1 ± 1.41^b^	7.0 ± 0.93 ^a^	0.05

^a,b,c^ Different superscripts within a row indicate significant differences (*p* < 0.05).

## Data Availability

The data presented in this study are available on request from the corresponding author. The data are not publicly available to preserve client confidentiality.

## References

[1] USDA (2015). Swine 2012 Part I: Baseline Reference of Swine Health and Management in the United States.

[2] Stalder K.J., Knauer M., Baas T.J., Rothschild M.F., Mabry J.W. (2004). Sow longevity. Pig News Inf..

[3] Deen J., Xue J. Sow mortality in the US: An industry-wide perspective. Proceedings of the Allen D. Leman Swine Conference.

[4] Anil S.S., Anil L., Deen J. (2009). Effect of lameness on sow longevity. J. Am. Vet. Med. Assoc..

[5] Anil S.S., Anil L.L., Deen J. (2005). Evaluation of patterns of removal and associations among culling because of lameness and sow productivity traits in swine breeding herds. J. Am. Vet. Med. Assoc..

[6] Boyle L., Lenard F.C., Lynch B., Brophy P. (1998). Sow culling patterns and sow welfare. Ir. Vet. J..

[7] Karriker L.A., Abell C.E., Pairis-Garcia M.D., Holt W.A., Sun G., Coetzee J.F., Johnson A.K., Hoff S.J., Stalder K.J. (2013). Validation of a lameness model in sows using physiological and mechanical measurements. J. Anim. Sci..

[8] Mohling C.M., Johnson A.K., Coetzee J.F., Karriker L.A., Stalder K.J., Abell C.E., Tyler H.D., Millman S.T. (2014). Evaluation of mechanical and thermal nociception as objective tools to measure painful and nonpainful lameness phases in multiparous sows. J. Anim. Sci..

[9] Pairis-Garcia M.D., Johnson A.K., Abell C.A., Coetzee J.F., Karriker L.A., Millman S.T., Stalder K.J. (2015). Measuring the efficacy of flunixin meglumine and meloxicam for lame sows using a GAITFour pressure mat and an embedded microcomputer-based force plate system. J. Anim. Sci..

[10] Tapper K.R., Johnson A.K., Karriker L.A., Stalder K.J., Coetzee J.H., Parsons R.L., Millman S.T. (2013). Pressure algometry and thermal sensitivity for assessing pain sensitivity and effects of flunixin meglumine and sodium salicylate in a transient lameness model in sows. Livest. Sci..

[11] Mohling C.M., Johnson A.K., Coetzee J.F., Karriker L.A., Abell C.E., Millman S.T., Stalder K.J. (2014). Kinematics as objective tools to evaluate lameness phases in multiparous sows. Livest. Sci..

[12] Pairis-Garcia M.D., Johnson A.K., Stalder K.J., Abell C.A., Karriker L.A., Coetzee J.F., Millman S.T. (2015). Behavioural evaluation of analgesic efficacy for pain mitigation in lame sows. Anim. Welf..

[13] Parsons R.L., Johnson A.K., Coetzee J.F., Karriker L.A., Mohling C.M., Pairis-Garcia M.D., Stalder K.J., Millman S.T. (2015). Sow behavioral responses to transient, chemically-induced synovitis lameness. Acta Agric. Scand. A—Anim. Sci..

[14] Roca A., Johnson A.K., Karriker L.A., Timms L.L., Abell C.E., Stalder K.J. (2016). How do sow postures change when lameness is induced using a chemical synovitis model?. Livest. Sci..

[15] Pairis-Garcia M.D., Karriker L.A., Johnson A.K., Kukanich B., Wulf L., Millman S.T., Stalder K.J., Coetzee J.F. (2013). Pharmacokinetics of flunixin meglumine in mature swine after intravenous, intramuscular and oral administration. BMC Vet. Rees..

[16] Pairis-Garcia M.D., Johnson A.K., Stalder K., Karriker L., Coetzee J., Millman S.T. (2014). Measuring the efficacy of flunixin meglumine and meloxicam for lame sows using nociceptive threshold tests. Anim. Welf..

[17] Manson F.J., Leaver J.D. (1988). The influence of concentrate amount on locomotion and clinical lameness in dairy cattle. Anim. Prod..

[18] Main D.C., Clegg J., Spatz A., Green L.E. (2000). Repeatability of a lameness scoring system for finishing pigs. Vet. Rec..

[19] Sprecher D.J., Hostetler D.E., Kaneene J.B. (1997). A lameness scoring system that uses posture and gait to predict dairy cattle reproductive performance. Theriogenology.

[20] Smith B. (1988). Lameness in pigs associated with foot and limb disorders. Practice.

[21] Wells G.A.H. (1984). Locomotor disorders of the pig. Practice.

[22] Federation of Animal Science Societies FASS (2010). Guide for the Care and Use of Agricultural Animals in Agricultural Research and Teaching.

[23] NRC (2012). National Research Council. Nutrient Requirements of Swine.

[24] NPB (2020). Transport Quality Assurance (V. 7).

[25] Nalon E., Maes D., Piepers S., Taylor P., van Riet M.M.J., Janssens G.P.J., Millet S., Tuyttens F.A.M. (2016). Factors affecting mechanical nociceptive thresholds in healthy sows. Vet. Anaesth. Analg..

